# Deep Neural Network-Based Visual Feedback System for Nasopharyngeal Swab Sampling

**DOI:** 10.3390/s23208443

**Published:** 2023-10-13

**Authors:** Suhun Jung, Yonghwan Moon, Jeongryul Kim, Keri Kim

**Affiliations:** 1 Artificial Intelligence and Robot Institute, Korea Institute of Science and Technology, 5, Hwarang-ro 14-gil, Seongbuk-gu, Seoul 02792, Republic of Korea; 024840@kist.re.kr; 2 School of Mechanical Engineering, Korea University, 145 Anam-ro, Seongbuk-gu, Seoul 02841, Republic of Korea; 3 Augmented Safety System with Intelligence Sensing and Tracking, Korea Institute of Science and Technology, 5 Hwarang-ro 14-gil, Seongbuk-gu, Seoul 02792, Republic of Korea; 4 Division of Bio-Medical Science and Technology, University of Science and Technology, 217 Gajeong-ro, Yuseong-gu, Daejeon 34113, Republic of Korea

**Keywords:** nasopharyngeal swab testing, load cell, fiducial marker, augmented reality, 1-dimensional convolution neural network

## Abstract

During the 2019 coronavirus disease pandemic, robotic-based systems for swab sampling were developed to reduce burdens on healthcare workers and their risk of infection. Teleoperated sampling systems are especially appreciated as they fundamentally prevent contact with suspected COVID-19 patients. However, the limited field of view of the installed cameras prevents the operator from recognizing the position and deformation of the swab inserted into the nasal cavity, which highly decreases the operating performance. To overcome this limitation, this study proposes a visual feedback system that monitors and reconstructs the shape of an NP swab using augmented reality (AR). The sampling device contained three load cells and measured the interaction force applied to the swab, while the shape information was captured using a motion-tracking program. These datasets were used to train a one-dimensional convolution neural network (1DCNN) model, which estimated the coordinates of three feature points of the swab in 2D X–Y plane. Based on these points, the virtual shape of the swab, reflecting the curvature of the actual one, was reconstructed and overlaid on the visual display. The accuracy of the 1DCNN model was evaluated on a 2D plane under ten different bending conditions. The results demonstrate that the x-values of the predicted points show errors of under 0.590 mm from $P_{0}$, while those of $P_{1}$ and $P_{2}$ show a biased error of about −1.5 mm with constant standard deviations. For the y-values, the error of all feature points under positive bending is uniformly estimated with under 1 mm of difference, when the error under negative bending increases depending on the amount of deformation. Finally, experiments using a collaborative robot validate its ability to visualize the actual swab’s position and deformation on the camera image of 2D and 3D phantoms.

## 1. Introduction

The recent respiratory epidemic, known as coronavirus disease 2019 (COVID-19), has highlighted the vulnerability of quarantine systems worldwide. To effectively cope with COVID-19, the World Health Organization has urged a diagnosis using bronchial specimens. Since then, COVID-19 testing has become a routine procedure that degrades quality of life, particularly for healthcare workers (HCWs) [1,2]. Many HCWs have been mobilized to test for COVID-19, and their frequent contact with subjects exposes them to aerosol infections [3,4]. To minimize the risk of aerosol infection, HCWs wear protective clothing and masks even during the harsh summer season. Therefore, HCWs work under harsh physical and mental conditions and have consistently complained about them.

Recent studies have focused on robotic-based systems for swab sampling to reduce the burden on HCWs and improve the working environment. First, the basic forms of these automated robots are designed to replace the sampling motion only [5,6,7]. However, these systems still require a significant human workforce for the preparation of tasks such as attaching, cutting, and disposing of swabs. To overcome these challenges, some studies have expanded the functionality of the robots so that they can replace most of the tasks [8,9,10,11]. These robotic systems are designed to automatically recognize the location of the suspected COVID-19 patients using camera vision and to follow the sampling path. In particular, the teleoperated system enables nasopharyngeal (NP) sampling, which is more difficult to achieve but has higher diagnostic accuracy than oropharyngeal (OP) sampling [12]. However, with confined images from the camera attached to the end of the manipulator [8,9,10,11,13], teleoperation can lead to incorrect sampling and may damage the nasal vestibule roof and septum, causing severe pain or bleeding [14,15]. 

There have been studies suggesting computer vision methodologies to obtain meaningful diagnosis information from medical images [16,17,18]. In particular, vision-based feedback technologies, such as localization, classification, and segmentation methods, are widely studied as they ease recognition intuitively [19,20,21]. Augmented reality (AR) refers to one of the techniques in computer vision that overlays virtual images on those of the real world. Several studies have attempted to provide images of 3D objects through AR, validating their performance in the fields of medical assistance and diagnosis [22,23,24,25]. Within the pre-installed markers and tracking conditions, AR provides fast and precise guidance in environments where targeted objects, such as swabs, are hidden behind other objects. Considering this, AR-based virtual images might deliver essential information about the deformed shape and location of the swab inserted into the nasal cavity. 

Estimating the exact shape of swabs is another obstacle to their visualization because the precise shape in contact with the nasal cavity remains unknown. A swab that responds to external forces can be modeled if it is regarded as a uniform rod [26,27]. However, calculation-based modeling that considers deformation within limited conditions requires complex calculations. Recently, machine learning technology has been proposed as a method to estimate a specific value from an arbitrary model without an exact understanding of the underlying mechanisms. Machine learning refers to a technology in which a certain model learns from given data. In addition, convolutional neural networks (CNN), which are widely known to detect the local characteristics of data, have been applied to nonlinear one-dimensional time-series data [28,29,30,31,32]. Furthermore, machine learning models that combine multiple sensor inputs have shown promising results in the prediction of nonlinear outputs [33,34]. Hence, owing to the loadcell data in this study containing time-series contact force data from multi-channel sensors, a one-dimensional CNN (1DCNN) with multiple filters is suitable for capturing concealed patterns within the measured force.

**Figure 1:**
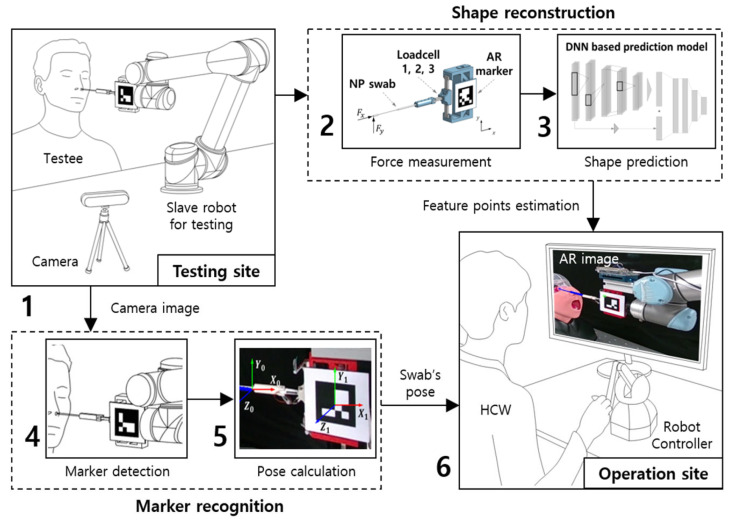
System overview of AR-based visual feedback system. (1) Nasopharyngeal sampling under automated environment. (2) Measuring contact force from the sampling device. (3) Estimating the exact location of feature points. (4) Recognizing markers from camera images while sampling. (5) Calculating the pose of the sampling device from the attached fiducial marker. (6) Delivering AR-based guides regarding the shape of the nasopharyngeal swab.

**Figure 2:**
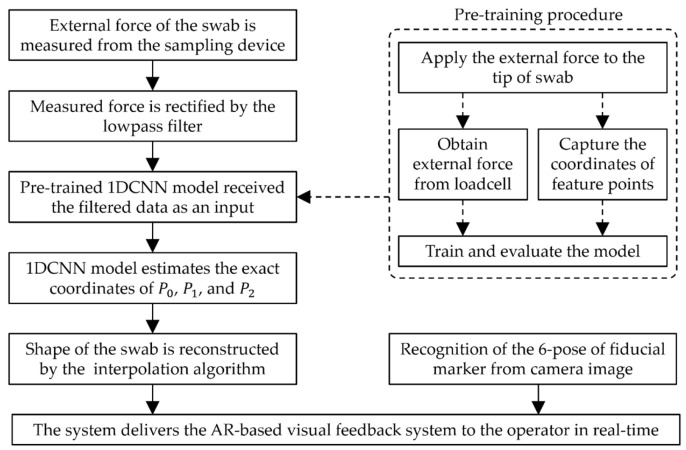
Flow chart of the research methodology.

This study proposes an AR-based visual feedback system that predicts the shape of an NP swab inserted inside the nasal cavity. As illustrated in Figure 1, the system generates the shape of the swab based on the contact force and fiducial markers and transmits these data to the operator via monitoring devices. The contact force applied to the swab was measured using a sampling device with three embedded load cells. The contact forces were synchronized with the coordinates of the feature points of the swab obtained using motion-tracking software. To determine the approximate shape of the swab, we adopted a 1DCNN-based structure. This model predicts the x- and y-coordinates of the three feature points using the rectified contact force as its input. The intermediate points, which comprised the continuous curve of the swab, were calculated from the estimation output. With a six-pose fiducial marker on the sampling device, the reconstructed shape of the swab was delivered to an AR environment. A flow chart of the research methodology given above is illustrated in Figure 2.

To evaluate the accuracy of the prediction model, we conducted a test under ten bending conditions in a two-dimensional (2D) sagittal plane. Furthermore, the errors and standard deviations of the feature points were evaluated. The 3D environment was designed to validate the effectiveness of the AR system for both types of phantoms. The results of the experiment with the 2D phantom showed a similarity between the estimated and actual swab shapes. The experiment with the three-dimensional (3D) phantom presented results regarding the shape and force of the swab in contact with the nasal cavity. 

The paper is organized as follows: Section 2 presents the methodologies for reconstructing the shape of a swab; Section 3 details the structure and training of the estimation model; and Section 4 presents the settings and results of the two experiments that verify the effectiveness of the estimation model and AR-based system. Finally, Section 5 presents the discussion and conclusions.

## 2. Reconstruction of Nasopharyngeal Swab

**Figure 3:**
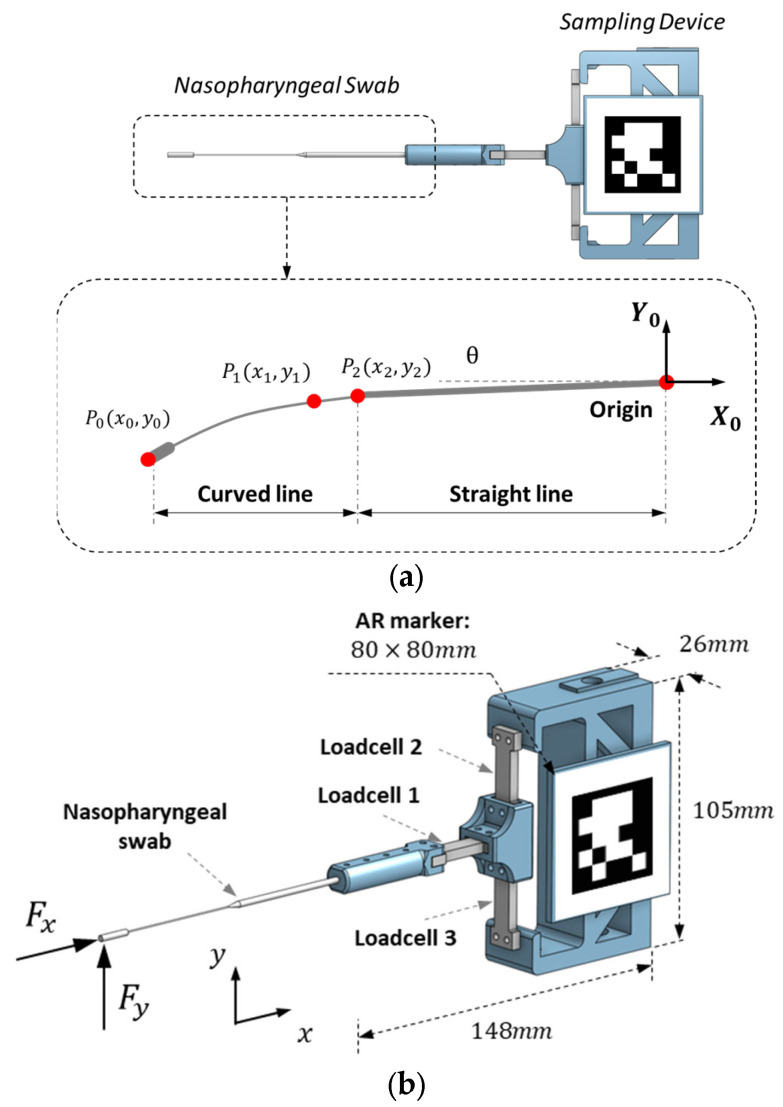
Modeling and apparatus for swab reconstruction. (**a**) Coordinate systems for feature points of nasopharyngeal swab and (**b**) configuration of the sampling device.

This section proposes modeling and visualization methods for NP swabs. As shown in Figure 3a, the NP swab consists of a rod with two distinct thicknesses for flexibility at the tip. The swab had a total length of approximately 150 mm, and the thick and thin parts had thickness of 2.5 mm and 0.95 mm, respectively. The bending of the swab was assumed to occur primarily on the thinner side, whereas the thicker side remained straight. Hence, the swab was modeled as a function using three feature points as inputs. A sampling device equipped with three load cells was developed to measure the contact force applied to the swab. The positions of the three feature points from the contact force were predicted using a machine learning model, the details of which are explained in Section 3. In addition, a fiducial marker attached to the sampling device was used to accurately position the reconstructed swab within the camera screen.

### 2.1. Methods for the Shape Reconstruction of NP Swab

The entire shape of the swab was reconstructed, including intermediate points between the three feature points $P_{0}$, $P_{1}$, and $P_{2}$. After calculating the shape in a 2D sagittal plane, the AR environment envisions a 3D model of the swab that reflects the translation, rotation, and bending of the 3D object. 

Figure 3b shows the axes and detailed configuration of the swab. The separation of the straight and curved lines considers the bending of the NP swab when a small (less than 5 gf) force in the y-direction is applied to the tip. A straight line connects the origin and $P_{2}$, when the origin is set at the junction of the swab and its holder. The curve between $P_{0}$ and $P_{2}$ is filled by the approximated points derived from the function based on second-order Lagrangian interpolation. This function, $f\left(x\right),$ is composed of the coordinates $P_{0}$, $P_{1}$, $P_{2}$, as presented in Equations (1) and (2).

(1) $$f\left(x_{k}\right)=\frac{\left(x_{k}-x_{1}\right)\left(x_{k}-x_{2}\right)}{\left(x_{0}-x_{1}\right)\left(x_{0}-x_{2}\right)}y_{0}+\frac{\left(x_{k}-x_{2}\right)\left(x_{k}-x_{0}\right)}{\left(x_{1}-x_{2}\right)\left(x_{1}-x_{0}\right)}y_{1}+\frac{\left(x_{k}-x_{0}\right)\left(x_{k}-x_{1}\right)}{\left(x_{2}-x_{0}\right)\left(x_{2}-x_{1}\right)}y_{2},$$

(2) $$x_{k}=x_{0}+\frac{k}{N}\left(x_{2}-x_{0}\right),\;where\;k=0,1,2\cdots N,$$

where $x_{0}$, $x_{1}$, $x_{2}$, $y_{0}$, $y_{1}$, and $y_{2}$ represent the x–y coordinates of $P_{0}$, $P_{1}$, and $P_{2}$ when $x_{k}$ is the x-coordinate of the intermediate points between $P_{0}$ and $P_{2}$. N indicates the number of points and determines the resolution of the curve.

The most important contact with the nasal cavity, which determines the completion of sampling, occurs along the anterior–posterior direction (*x*-axis in this study), that is, perpendicular to the swab. However, the dominant interaction force that determines the shape of the swab in contact with the nasal cavity is the force along the vertical direction (*y*-axis in this study). In this study, a sampling device was developed to monitor the contact force applied to the tip along both the x- and y-axes and to deliver a virtual guide to the approximate shape. The configuration of the sampling device is illustrated in Figure 3b. Three loadcell units (HT Sensor Technology, Shaanxi, China) were installed on the sampling device to measure contact forces in the sagittal plane. When only load cell 1 is positioned for the force in the y-direction, load cells 2 and 3 are responsible for measuring the force in the x-direction. This structure allows the distinction of the interaction force by subtracting the measured forces from load cells 2 and 3. In addition, this structure shortens the distance between the base and load cell 1, thereby diminishing high-frequency noise induced by vibration and friction. Furthermore, a frontal part connected to load cell 1 was designed to grip 40 mm of the NP swab for convenient sampling and replacement. The small size of the sampling device (148 mm × 105 mm × 26 mm) enabled the fixation of robot manipulation and hand-carried sampling.

### 2.2. Data Acquisition and Filtering Method of the Applied Force and Motion

**Figure 4:**
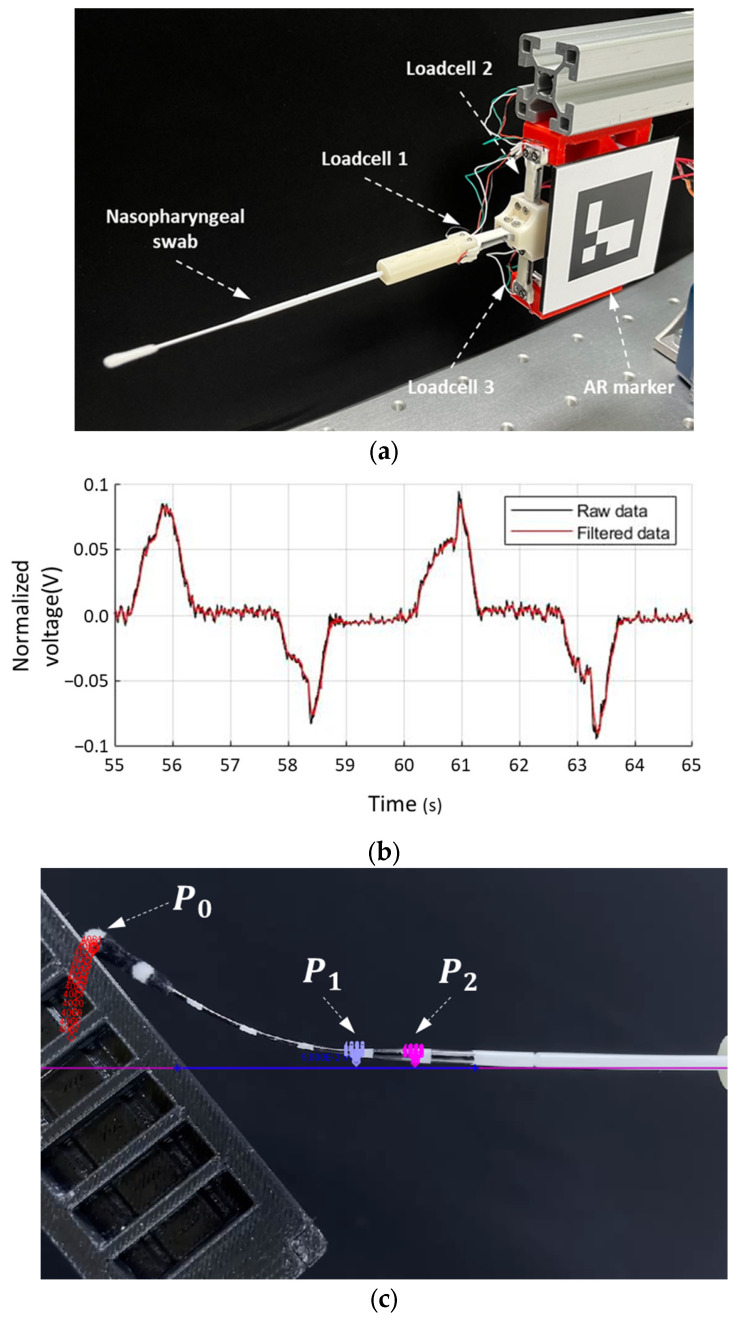
Data acquisition method from loadcell and tracking program. (**a**) Device setting for measurement; (**b**) raw and rectified loadcell data; and (**c**) three tracking points of nasopharyngeal (NP) swab.

Figure 4a shows the setting of the sampling device to simultaneously measure the shape and contact force when bending of the swab occurs owing to external contact. To measure the loadcell data, a USB-type data acquisition module (USB-6003, National Instruments, Austin, TX, USA) acquired analog signals from the load cell at a 1 k sampling frequency. A first-order low-pass filter (LPF) was used to rectify the electrical and frictional noise. Figure 4b shows the acquired raw data (black line) and the filtered data (red line). This LPF was applied equally to the tracking data of the feature points representing the shape of the swab. These rectified load cells and tracking data were used as datasets to train the machine learning model. The LPF equation is as follows:

(3) $${\bar{x}}_{k}=\tau /(\tau +∆t){\bar{x}}_{k-1}+∆t/(\tau +∆t)x_{k},$$

where $x_{k}$ is the measured sensor data of time k, and ${\bar{x}}_{k}$ and ${\bar{x}}_{k-1}$ refer to the filtered sensor data of time k and k−1, respectively. τ denotes the time constant of the filter, and ∆t is the sampling period.

To obtain information regarding the bending swab in the sagittal plane, a video analysis tool (Tracker 5.1.5, Open Source Physics, USA) was used to track the feature points on marked NP swabs. This program enabled the tracking of the positions of matching images with the preset image template. Figure 4c shows the tracking points of the three feature points $P_{0}$, $P_{1}$, and $P_{2}$. The three feature points were selected to represent the bending part among the points on the swab at 10 mm intervals. $P_{0}$ represents the tip of the swab, which was the most sensitive part. $P_{2}$ was located at the starting point of the narrowing of its diameter when the $P_{1}$ moved 10 mm to the tip from $P_{2}$. The images (red dots for $P_{0}$) show the tracked history of the previous frames regarding the points. The coordinates of the three points were synchronized with the measured loadcell data and resampled to ensure the same dataset size. 

### 2.3. Visualization in Real-Time Augmented-Reality Model

**Figure 5:**
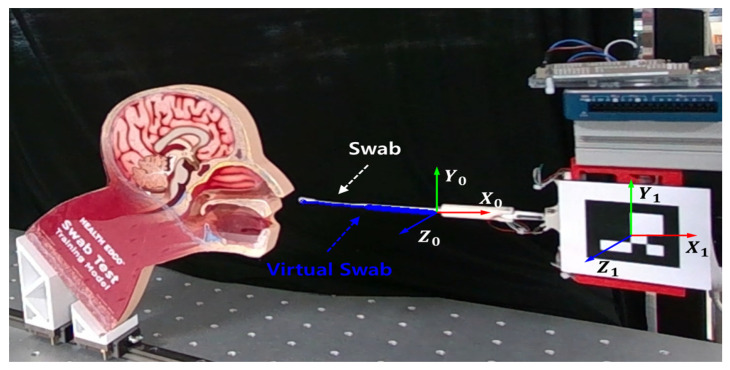
Reconstructed swab on the camera image. NP swab (white) is overlain with the virtual swab (blue) attached to the end of the swab holder (coordinate system 0).

By applying the machine learning method described in Section 3 and using the measured data, three points, $P_{0}$, $P_{1}$, and $P_{2}$, can be predicted using the contact force. To visualize these points in a 3D virtual space, a fiducial marker of size 80 mm × 80 mm was used to identify the real-world location, orientation, and scale of the model. A custom application based on the ArUco marker fiducial system was used to merge the 3D model with videos from an RGBD camera (RealSense D455, Intel Corp., Santa Clara, CA, USA). Figure 5 shows the AR for NP swab samplings. The system performs automated registration of the swab model by placing the approximated points on the swab using the six-degrees-of-freedom (6-DoF) pose of the marker. The AR system is divided into separate subsystems for computation and visualization, such that time delays can be minimized. In addition, user datagram protocol (UDP) communication was adopted to deliver information about the estimated feature points between the two subsystems. Consequently, the UDP can send the predicted feature points to the visualization system once every 40 ms, with a delay of less than 1 ms.

## 3. Estimation Model for Shape Reconstruction

This section proposes a machine learning model that predicts the positions of feature points using measurement data from multiple sensors. The estimation model consists of 1DCNN layers, which are one of the most widely used deep neural networks. The following multilayer perceptron (MLP) calculates the final output, reflecting the nonlinearity of the dataset. The training dataset was acquired using the methodology described in Section 2.2. Using the estimation data, feature points were drawn in the 2D plane for comparison with the ground-truth data.

### 3.1. Basic Structure of 1DCNN Model

**Figure 6:**
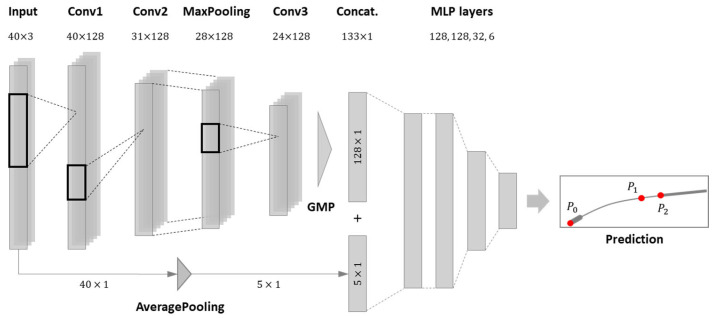
Basic structure of the custom 1DCNN network.

The structure of the estimation model is composed of a 1DCNN and an MLP, as shown in Figure 6. The three-channel measured data were reshaped into a time-series input with a window size of 40 ms. After this input is delivered to the CNN layers, a global max pooling (GMP) layer extracts the maximum features of the filtered data. The GMP reflects the general tendency of multiple sensors and diminishes their dimensions. As load cell 1 is the dominant observer of the vertical force, the residual connection provides a detour for the undeterred information of load cell 1 after passing through an average-pooling layer. In addition, a concatenate layer merges the data from the CNN and residual layer. Subsequently, the concatenated data were sent to an MLP, stacking multiple layers to reflect the nonlinearity of the dataset. The final output contains information about the exact coordinates of the feature points $P_{0}$, $P_{1}$, and $P_{2}$. In addition to the last layer of the MLP, a rectified linear unit was adopted as an activation function for the CNN and MLP layers. Furthermore, a hyperbolic tangent function was chosen as the last function for nonlinearity. 

### 3.2. Training of the Model

The training dataset was prepared from repetitive bending tests using the developed sampling device and a camera for image tracking. After an object with arbitrary inclination was loaded into a linear guide, it moved horizontally and contacted the tip of the NP swab. The contact force deformed the swab in arbitrary directions, and the apparatus measured the bending force and shape. These data were rectified using the filter explained in Section 2.2 and then divided into training, evaluation, and test datasets at a 6:2:2 ratio.

**Figure 7:**
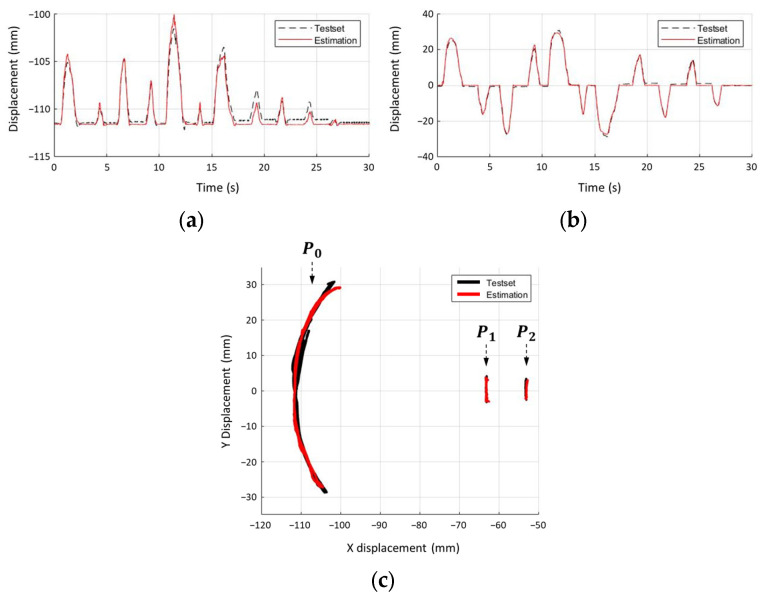
Comparison between test sets and predictions. (**a**) Estimated x-data of $P_{0}$, (**b**) estimated y-data of $P_{0}$, and (**c**) estimated feature points in the 2D plane.

For the training procedure, the mean square error (MSE) was used as a loss function, and the adaptive moment estimation (Adam) was used as an optimizer. The early stopping function, which incorporated patience, monitored and saved the trained model while reaching its limit of improvement. When the training and validation losses reached 5.1621 × 10^−5^ and 1.0462 × 10^−4^, respectively, the training procedure was stopped after seven epochs. The root MSEs of the three feature points were calculated as 0.4927, 0.1589, and 0.0968 for x and 0.0620, 0.7986, and 0.1069 for y. Figure 7a,b shows the training and estimated x- and y-values of the feature point $P_{0}$, while the 2D locus of point $P_{0}$ is shown in Figure 7c. The predicted points (red dots in Figure 7c) appear to be less disturbed by external noise than the raw tracking data (black dots in Figure 7c).

## 4. Experimental Setups and Results

### 4.1. Experiment with Phantom Models

**Figure 8:**
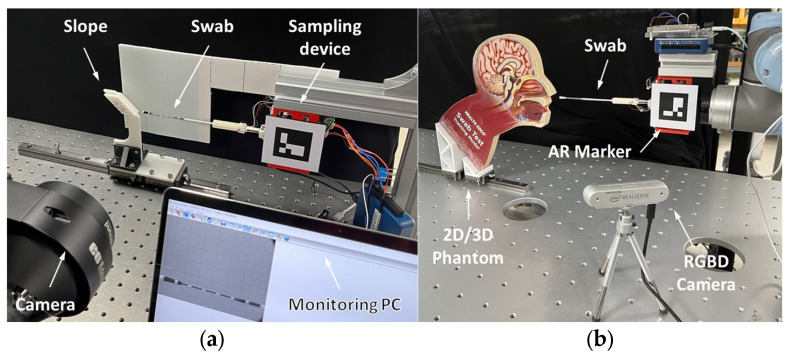
Experimental environments of accuracy and performance evaluation. (**a**) Assessment of accuracy on 2D plane and (**b**) validation test using the 2D/3D phantom.

Two types of experiments were conducted to simulate the NP sampling situation and evaluate the accuracy and performance of the shape reconstruction system. Figure 8a shows the sampling environment for the evaluation of the estimation accuracy of the feature points in the 2D sagittal plane. Based on ten bending conditions at ±5, ±10, ±15, ±20, and ±25 mm of y displacement, the contact forces and swab shapes were recorded from 20 sets of repetitive contacts with a custom slope. The positions of the feature points were measured using a monochrome camera (EO-1312, Edmund Optics, Barrington, NJ, USA) attached to a telecentric lens (58259, Edmund Optics, Barrington, NJ, USA). This system captures a corrected image from the camera, allowing an accurate analysis of coordinates that are free from distortion. In addition, the predicted coordinates of the feature points were compared with the ground-truth data. 

The system performance was validated in 3D environments and imitated actual sampling conditions. Figure 8b presents the experimental setup for the 3D sampling experiment. Both 2D and 3D phantoms were used as substitutes for patients suspected of being infected. The transparent cover of the 2D phantom allowed the interior of the nasal cavity to be visualized. Despite the invisibility of the internal structure of the 3D phantom model, the AR model of the swab helps to evaluate the approximate shape and location in real time. The swab was inserted using an UR5 robot arm (Universal Robots, Inc., Odense, Denmark) to emulate a remote sampling environment. Three cases of insertion were supposed to reflect various deformations: (1) normal sampling (0°), (2) excessive contact with the upper nasal cavity (30–40° range), and (3) excessive contact with the lower nasal cavity (−20 to 30° range). An NP swab (SG Medical, Seoul, Republic of Korea) was fixed to the swab holder after insertion at 40 mm. The RGBD camera tracked the fiducial marker to visualize the AR images using its position and orientation. The final AR images were delivered to the monitor and recorded for each sampling sequence. Furthermore, for every case in the experiments, each sequence took approximately 910 s.

### 4.2. Validation of Accuracy and Performance of Estimation System

**Figure 9:**
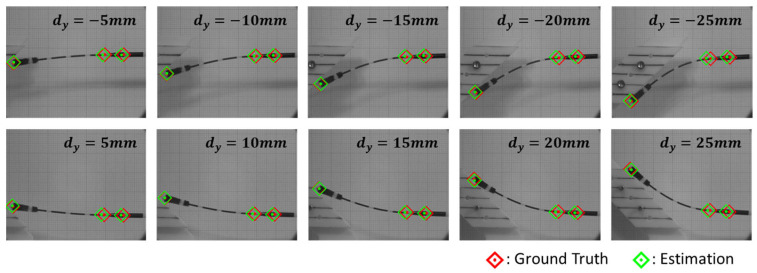
Ten bending conditions for the 2D accuracy experiment.

Figure 9 shows the 2D sampling experiments under ten bending conditions. The error and standard deviation of each feature point were calculated from the measured data, as presented in Table 1. The results showed that the predicted points were biased toward the x-coordinates with a small distribution. In particular, the biased error of $P_{1}$ and $P_{2}$ (error≅−1.5mm) was larger than that of $P_{0}$ ($\left|error\right|<0.5mm$), which was as attributed to the influence of the initial position setting of the feature points. The results for the y-coordinate showed distinct trends in the positive (5–25 mm) and negative (−5 to 25 mm) bending directions. A uniform error of less than 1 mm was observed in the positive bending direction. Conversely, the error in the negative direction increased with the amount of deformation. However, the same level of standard deviation was observed in both directions. Owing to the tip of the swab being subjected to the largest displacement, the largest error was observed at the feature point $P_{0}$. The average positions of the ground truth of the three feature points (red diamonds with centered dots) and the estimation points (green diamonds with centered dots) are marked in Figure 9 to visualize the error.

**Figure 10:**
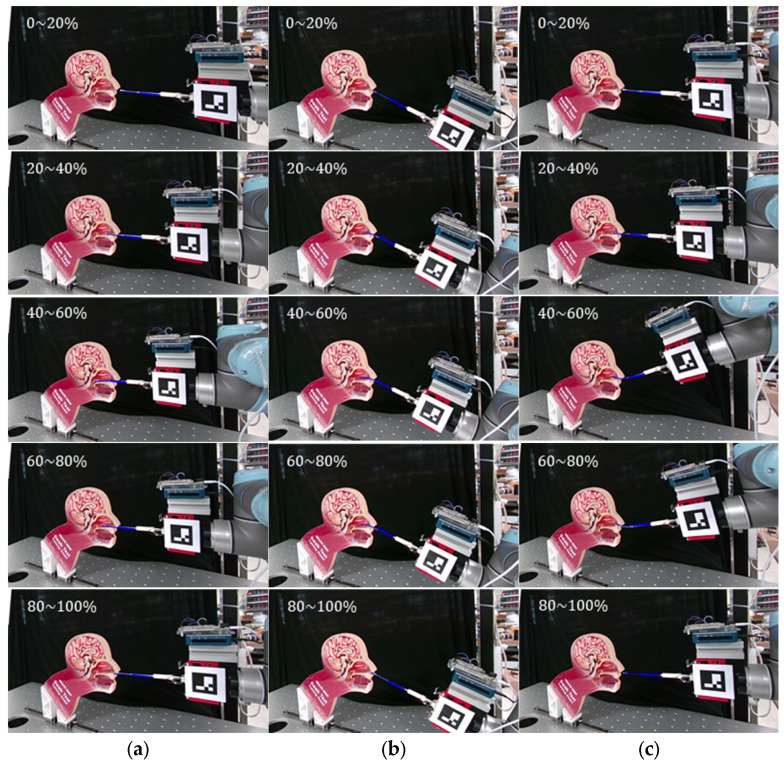
Evaluation using 2D sagittal phantom model. (**a**) Successful sampling, (**b**) excessive contact with upper side of the nasal cavity, and (**c**) excessive contact with lower side of the nasal cavity.

**Figure 11:**
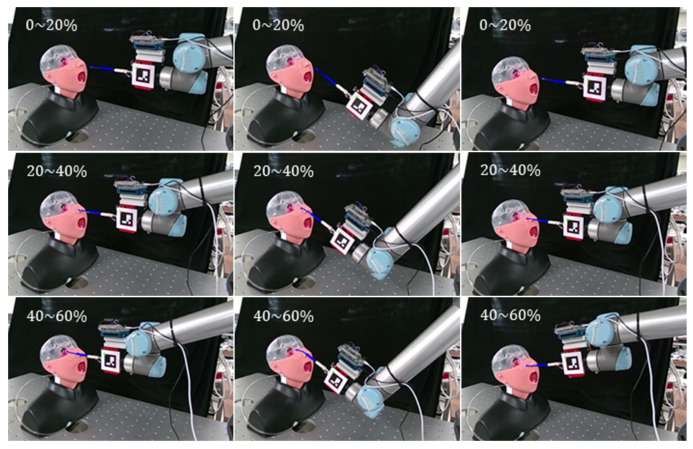

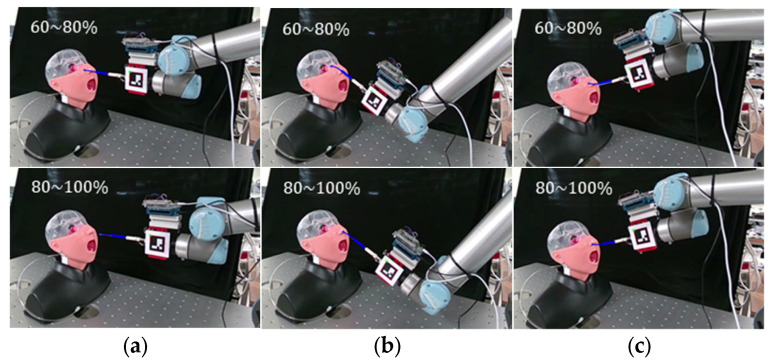
Evaluation using 3D phantom model. (**a**) Successful sampling, (**b**) excessive contact with upper side of the nasal cavity, and (**c**) excessive contact with lower side of the nasal cavity.

The results for the sampling situations for the 2D and 3D models are shown in Figure 10 and Figure 11, respectively. The AR images after registration (blue line in the images) are described according to the sampling progress. Based on the experimental results on the 2D sagittal phantom in Figure 10, the virtual swab was compared with an actual NP swab bent inside the nasal cavity. Assuming successful sampling (Figure 10a), the virtual swab began to deform when the tip started to contact the posterior NP wall. The actual and predicted shapes of the swab (white and blue images, respectively) appeared to have similar degrees of bending along the negative direction. In addition, Figure 10b,c represent the excessive contact situations with the upper and lower sides of the nasal cavity due to operational errors. When the swab contacted the inferior nasal concha and hard palate of a rigid 2D phantom (40–80% of (b) and (c) in Figure 10), excessive bending occurred equally for the AR model and real swab in both cases.

Figure 11 shows the procedure of the NP sampling experiment for the 3D phantom. The experiments were performed according to three scenarios identical to the conditions of the 2D phantom experiments. In a successful sampling situation (Figure 11a), the swab passed straight through the nasal cavity and contacted the NP wall. Because the nasal cavity was made of soft material, the swab formed an almost straight line with slight bending. Conversely, the swab was deformed by contact with the upper and lower parts of the nasal cavity (Figure 11b and Figure 11c, respectively).

**Figure 12:**
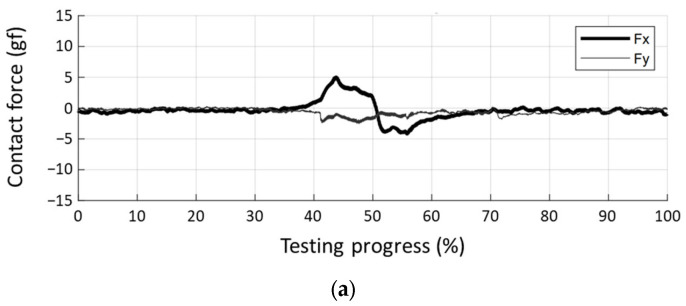

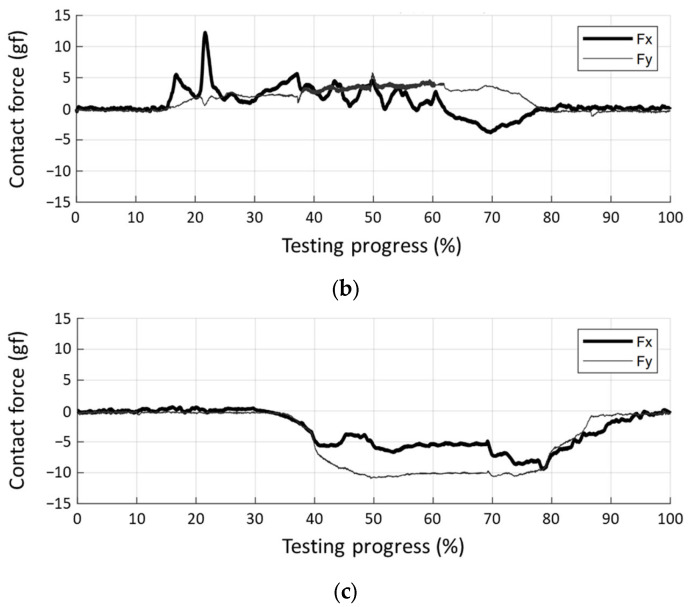
Contact force of swab with nasal cavity of 3D phantom. (**a**) Successful sampling, (**b**) excessive contact with upper side of the nasal cavity, and (**c**) excessive contact with lower side of the nasal cavity.

The contact force and reconstructed shape of the NP swab entering the 3D phantom are presented in Figure 12 for the three scenarios. The contact force measured in the x- and y-directions using load cells attached to the sampling device was plotted for a single sampling epoch. In the successful sampling shown in Figure 12a, the y-direction force was within 3 g with minimal contact with the nasal cavity. The x-direction force was positive and negative before and after reaching the deepest insertion area (approximately 50% of the progress). This seems to be caused by friction acting in a direction opposite to the movement of the swab. During sampling with excessive contact, a relatively large y-direction contact force was observed. In particular, a strong contact with the top side of the interior (Figure 12b) resulted in a large x-direction force. Conversely, contact with the bottom side (Figure 12c) generated mostly a negative x-direction force. This is because the swab contact mostly occurred during withdrawal (after 40% of progress) after the deepest insertion.

**Figure 13:**
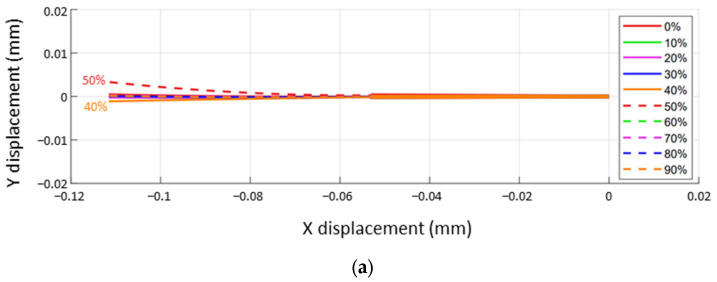

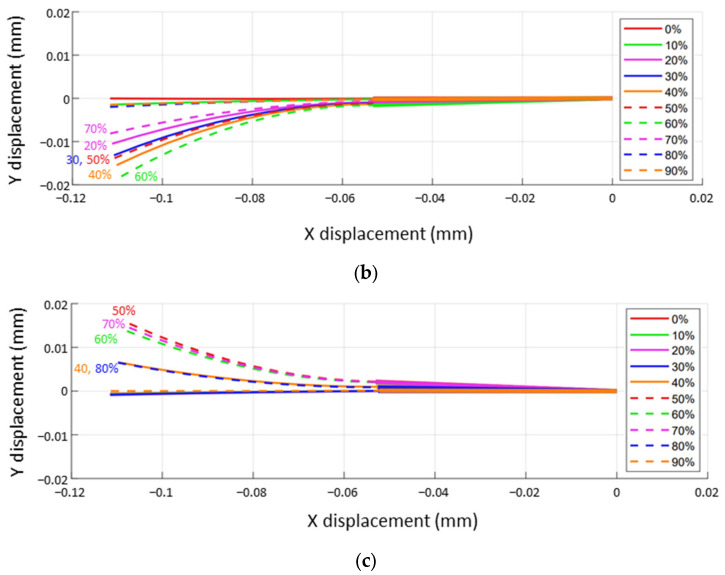
Reconstructed shape of the inserted swab. (**a**) Successful sampling, (**b**) excessive contact with upper side of the nasal cavity, and (**c**) excessive contact with lower side of the nasal cavity.

Figure 13 shows the reconstructed shape of the swab within the 3D phantom based on the predicted points. Each shape was distinguished by the brightness of the lines according to the levels of sampling progress. For successful sampling shown in Figure 13a, most of the shapes remained straight because a slight contact force occurred in the y-direction. As shown in Figure 13b, when the contact in the upper part was excessive, the tip of the swab was bent in the negative y-direction. In Figure 12b, swab bending occurred during 20–70% of the sampling progress, where large contacts in the y-direction were made. For the excessive contact at the bottom in Figure 13c, the bending in the positive y-direction occurred during 40–80% of the progress owing to contact with the interior.

## 5. Discussion and Conclusions

This study proposes an AR-based visual feedback system that can deliver the approximate shape of an NP swab while hidden inside a nasal cavity. The NP swab was regarded as a combination of a straight rigid rod and a bending rod. Three feature points that could represent the swab shape were determined. The positions of the feature points and the contact force applied to the tip of the swab were measured using an RGBD camera and a sampling device equipped with three load cells. Using the coordinates of the feature points obtained from the motion-tracking software, the contact force formed a dataset for training. To predict the swab shape from the contact force, a 1DCNN-based estimation model was trained. The final shape of the continuous swab was calculated based on the predicted coordinates, and the visual feedback system provided information about the depth and deformation of the swab based on the six-pose fiducial marker. 

The results of the accuracy test on the 2D plane showed that biased errors occurred uniformly in the x-coordinates of all the feature points. The error on the x-values of the predicted points was under 0.590 mm for $P_{0}$, while the x-values of $P_{1}$ and $P_{2}$ show a biased error of about −1.5 mm. We found that the initial settings related to the swab insertion position and camera placement resulted in tracking errors for the feature points. The analysis data of the y-coordinates showed distinct results, depending on their direction. For bending in the positive direction, a relatively small error within 0.5 mm was observed. Conversely, the error in the negative direction increased with the degree of bending. This error was considered to be due to an internal gravity-related force from the structure of the sampling device, which affected the sensitivity of the load cells. Overall, the results of the 2D bending test demonstrated the ability to generate a virtual model by substituting the actual shape of the swab in real-time. 

Furthermore, the performance of the visual feedback system was evaluated through experiments using 2D and 3D phantoms. From the experiment of sampling the 2D phantom (Figure 10), the integrated system reconstructing the shape of the swab was evaluated by comparing real and virtual swabs. The shape of the virtual swab, bent by contact with the interior of the phantom, was approximately the same as that of the real swab. In particular, the results of the experiments assuming excessive contact (Figure 10b,c) showed the possibility of a visual feedback system to identify the potential risks of harsh contact with the nasal cavity in advance. In addition, the same experiment was conducted on the 3D phantom (Figure 11) to evaluate the feasibility of the system. Because the 3D phantom hindered the motion of the swab, the internal force and predicted shapes were measured and analyzed. Based on the results of measuring the contact force (Figure 12), two types of forces were observed: (1) bi-directional contact force in the x-direction during the insertion and withdrawal of the swab and (2) contact force in the y-direction that resulted in swab bending. Compared with the successful insertion situation, the insertion into the dangerous area showed large forces in the x- and y-directions by maintaining a relatively long contact time. While hidden inside the phantom, the reconstructed shape of the swab visually feeds back the bending, particularly at excessive contact at the top (20–70% of sampling progress in Figure 12b and Figure 13b) and the bottom (40–80% of sampling progress in Figure 12c and Figure 13c). 

Although other automated sampling systems have tried to optimize the position of the camera, the limited field of view (FOV) hidden behind the face increases the difficulty of operation [8,9,10,11,12]. The developed system generates a virtual swab within the visually inaccessible area, overcoming the limitations of previous studies. However, the current system is established under the assumption that the operators can have a comprehensive understanding of the exact swab’s pose in 3D based on the displayed 2D images. Considering that HCWs typically utilize both visual and sensory information simultaneously in the ordinal sampling process, this system also needs extension towards multisensory feedback. Further studies are required to evaluate the potential applicability of other types of visual feedback systems in nasopharyngeal (NP) swabs. A graphical interface indicating the direction and magnitude of the force applied to the swab might enhance the operator’s perception. Performance validation in vivo tests will provide insights into the clinical applications of the developed system. Currently, progress is underway to obtain ethical approval for the systematic evaluation of human subjects. The emotions of the subjects, such as psychological stability and anxiety, are also promising observational factors for the validation of the system.

## Figures and Tables

**Table 1:** Error between ground truth and predicted feature points.

	Mean of Error (mm)						Standard Deviation (mm)					
y Diff (mm)	$x_{0}$	$y_{0}$	$x_{1}$	$y_{1}$	$x_{2}$	$y_{2}$	$x_{0}$	$y_{0}$	$x_{1}$	$y_{1}$	$x_{2}$	$y_{2}$
25	0.522	−0.410	−1.191	−0.046	−1.506	0.064	0.164	0.334	0.035	0.056	0.015	0.036
20	0.202	−0.728	−1.435	−0.101	−1.646	0.007	0.086	0.155	0.019	0.028	0.014	0.038
15	0.198	−0.251	−1.269	0.024	−1.397	0.106	0.064	0.363	0.015	0.042	0.012	0.040
10	−0.125	0.236	−1.331	−0.059	−1.405	−0.036	0.055	0.111	0.017	0.032	0.008	0.031
5	−0.313	0.303	−1.350	−0.065	−1.391	−0.009	0.046	0.069	0.024	0.034	0.021	0.038
−5	0.066	0.473	−1.356	0.187	−1.550	0.151	0.109	0.233	0.032	0.043	0.010	0.028
−10	0.054	1.289	−1.406	0.200	−1.585	0.170	0.041	0.169	0.017	0.026	0.010	0.020
−15	−0.244	2.610	−1.456	0.363	−1.666	0.339	0.150	0.269	0.014	0.050	0.013	0.038
−20	−0.519	3.180	−1.552	0.408	−1.415	0.238	0.092	0.178	0.010	0.029	0.014	0.039
−25	−0.590	2.451	−1.821	0.250	−1.341	0.142	0.256	0.327	0.186	0.116	0.034	0.101

## Data Availability

Not applicable.

## Abbreviations

COVID-19: Coronavirus disease 2019, HCWs: health care workers, NP: nasopharyngeal, OP: oropharyngeal, AR: augmented reality, CNN: convolutional neural networks, 1DCNN: 1-dimensional convolution neural network
